# Mapping the Status of Healthcare Improvement Science through a Narrative Review in Six European Countries

**DOI:** 10.3390/ijerph16224480

**Published:** 2019-11-14

**Authors:** Manuel Lillo-Crespo, Maria Cristina Sierras-Davó, Alan Taylor, Katrina Ritters, Aimilia Karapostoli

**Affiliations:** 1 Nursing Department and International Mobility Coordinator, Faculty of Health Sciences, University of Alicante, Carretera de San Vicente del Raspeig s/n, 03690 San Vicente del Raspeig, Alicante, Spain; 2 Nursing Department, Faculty of Health Sciences, University of Alicante, Carretera de San Vicente del Raspeig s/n, 03690 San Vicente del Raspeig, Alicante, Spain; 3 Department of Social, Therapeutic and Community Studies, Coventry University, Gosford St, Coventry CV1 5DL, UK; 4 Coventry University, Gosford St, Coventry CV1 5DL, UK; 5 Department of Nursing, University of Peloponnese, Tripoli 22100, Greece

**Keywords:** healthcare improvement science, quality improvement, narrative review, patient-centered care, consensus statement, health education

## Abstract

With the aim to explore how improvement science is understood, taught, practiced, and its impact on quality healthcare across Europe, the Improvement Science Training for European Healthcare Workers (ISTEW) project “Improvement Science Training for European Healthcare Workers” was funded by the European Commission and integrated by 7 teams from different European countries. As part of the project, a narrative literature review was conducted between 2008 and 2019, including documents in all partners’ languages from 26 databases. Data collection and analysis involved a common database. Validation took place through partners’ discussions. Referring to healthcare improvement science (HIS), a variety of terms, tools, and techniques were reported with no baseline definition or specific framework. All partner teams were informed about the non-existence of a specific term equivalent to HIS in their mother languages, except for the English-speaking countries. A lack of consensus, regarding the understanding and implementation of HIS into the healthcare and educational contexts was found. Our findings have brought to light the gap existing in HIS within Europe, far from other nations, such as the US, where there is a clearer HIS picture. As a consequence, the authors suggest further developing the standardization of HIS understanding and education in Europe.

## 1. Introduction 

Currently, across Europe, an estimated 8–12% of hospitalized patients suffer adverse events, such as nosocomial infections, whilst receiving healthcare. Almost half of those adverse events could be avoided [1]. Therefore, the contribution of healthcare improvement methods and the science of implementing effective change in clinical practice to improve the delivery of efficient, effective, and high-quality services, with a focus on patient-centered care, is increasingly recognized as being an essential component of the healthcare worker’s role. Traditional efforts to detect adverse events have focused on voluntary reporting and the tracking of errors in health organizations. However, public health researchers have established that only 10 to 20 percent of errors are ever reported and, of those, 90 to 95 percent cause no harm to patients. Health institutions need a more effective way to identify events that do cause harm to patients in order to quantify the degree and severity of harm, and to select and test changes to reduce harm [2].

Education for multidisciplinary healthcare professionals covers a wide spectrum from undergraduate to postgraduate levels, including both online and face-to-face taught programmes. However, as Skela-Savic et al. have stated, healthcare improvement science (HIS) and its education were not equally developed in all European countries [3]. In fact, in countries like Spain the term does not have a meaning if it is used literally, while in Romania the concept exists, even though it has not been developed further. However, in Scotland, HIS is well known due to the Anglo-Saxon literature published mainly in the US, and is even a priority in education, policy, and healthcare. So, as a consequence, there is an educational need for developing agreed and standardized interprofessional study programmes and other materials in order to address this gap in the European curricula, ensuring that improvement science becomes a core competency for all healthcare graduates, as future healthcare staff will potentially have a direct impact on health systems and the quality of care for patients, contributing to reducing adverse patient events [4].

The Improvement Science Training for European Healthcare Workers project (ISTEW) has worked to fill the gap. In 2013, the project, funded by the European Commission and composed of seven countries working in partnership, aimed to develop accredited evidence-based improvement science education for healthcare professionals in Europe through an all-partner consensus [4]. The overall activities were a literature review about the topic, a scoping exercise of professional competencies and capabilities required to practice HIS, an educational gap analysis, a specific HIS evaluation framework, and the development of a consensus definition of HIS [3,5], also to create four accredited, interdisciplinary learning modules based on experiential HIS learning pedagogies [6]. Working with a multidisciplinary team of healthcare workers, educators and health services researchers from Italy, Poland, Romania, Slovenia, Spain, England, and Scotland, the project aimed to develop a collective understanding of HIS and the knowledge, tools, and techniques required to extend it. 

One of the ISTEW project’s main targets was to develop a consensus definition of HIS. Referred to as the ‘Bled definition’, after the place in which we reached consensus, a definition was agreed by over 80 HIS experts from across Europe, using a modified Delphi technique. They defined HIS as: “The generation of knowledge to cultivate change and deliver person-centred care that is safe, effective, efficient, equitable, and timely. It improves patient outcomes, health system performance, and population health” [3].

Moreover, the four training modules created were “Systems Thinking, Models for Improvement, Measurement for Improvement, and Managing Change and Communication in Healthcare” [6]. Since the project ended in 2015, work has continued, with the aim of developing a common HIS culture across Europe with specific education. Such is the case of the International Summer Programme on HIS, carried out by the University of Alicante (Spain) every year since 2016, in which the ISTEW modules’ contents have been applied [7]. Furthermore, the HIS evaluation framework created in the ISTEW has been piloted in more European and non-European contexts [5].

In this article we describe the literature review carried out since the early stages of the project to gauge the current level of consensus, understanding, practice, and education improvement science in healthcare education and practice, and those synonyms related to or understood as HIS across all partner countries until nowadays. We also draw on learning from other ISTEW project activities that considered the understanding and teaching of HIS across partner countries in more detail, and with a wider remit than searching available literature. [3,4,6,8,9,10,11].

## 2. Materials and Methods

### 2.1. Aim and Design

We undertook a structured literature review, answering four questions to map the meaning and understanding of Healthcare Improvement Science in different European contexts: How is Healthcare Improvement Science interpreted? How is Healthcare Improvement Science practised? How are multidisciplinary health students educated in (the various components of) Improvement Science? What is the impact of education in Healthcare Improvement Science on health organisations?

An integrative narrative literature review method was chosen, with the aim to describe and discuss the state of the science of the specific topic or theme selected from a theoretical and contextual point of view [12,13]. A critical analysis of the literature published in books and electronic or paper-based journal articles regarding healthcare improvement science was developed by the different partner teams participating in the ISTEW project, and later by this manuscript’s co-authors. Our purpose for using an integrative narrative literature review was to provide readers with up-to-date knowledge about HIS across Europe nowadays, elucidating what could potentially contribute to build science regarding the topic and inform research, practice, and policy initiatives [14]. However, this type of review does not describe the methodological approach that would permit reproduction of data nor answer to any specific quantitative research questions, as the topic has not been developed equally and broadly in the European context. A descriptive and narrative approach was used.

About the design, all partners followed a search protocol, specifying the search terms that were developed by the English team, working with local librarians to identify the most relevant databases for their countries (Appendix A).

Searches were carried out in early 2014 and updated in 2019, covering articles written in the previous ten years since the beginning in 2014 and therefore resulting in a total time period between 2004 and 2019. In addition to English, articles in other partners’ languages were covered. We found limited publications in the Italian, Slovene, and Spanish literature, which limited the research itself. The Romanian team restricted their search to articles in English but referred only to healthcare systems in Romania. The results of each partner countries literature search were then shared with the wider team in English, including partners’ narratives used through the analysis, in order to achieve a consensus focusing on answering the main questions established in the beginning.

### 2.2. Search Methods and Outcome

Data collection and analysis involved two stages. Stage one involved a search and compilation of the results per team through a narrative and integrative literature review using a common Excel database document. This database was standardised across all partner countries and gave details of sources and relevant article abstracts. Details such as the date of access, author, title, year of publication, type of study, source, or the abstract were included in that Excel document. The second stage was to write a summary of evidence per team from the database and surrounding articles (including relevant ‘grey’ literature sources) to answer each of the research questions. All partners participating decided who was the most appropriate from their teams to develop those activities according to their expertise and profile. During the research time period, online and face-to-face meetings took place to reach consensus through focus groups.

In total, 26 databases were searched by the partner countries, with the number of databases in each country search varying from three to ten. There was some overlap between partners searching the same database for country-specific articles, particularly in relation to Cochrane, PubMed, Emerald, Cinahl, and ProQuest. The databases searched by partners were: Academic Search Complete; BIDS—EBSCO Discovery Sapienza; BMJ Journals; Business Source Complete; CEEOL (Central and Eastern European Online Library); Cinahl; Cobiss; Cochrane; EBSCO Academic Search Complete; Embase; Emerald; GIMBE; Google Scholar; HMIC; ISI; Medline; Ovid Medical; Oxford Journals; ProQuest; PsycINFO; PubMed; SAGE Journals; Science Direct; Scopus; Springerlink; Wiley.

### 2.3. Criteria for Inclusion

**Figure 1:**
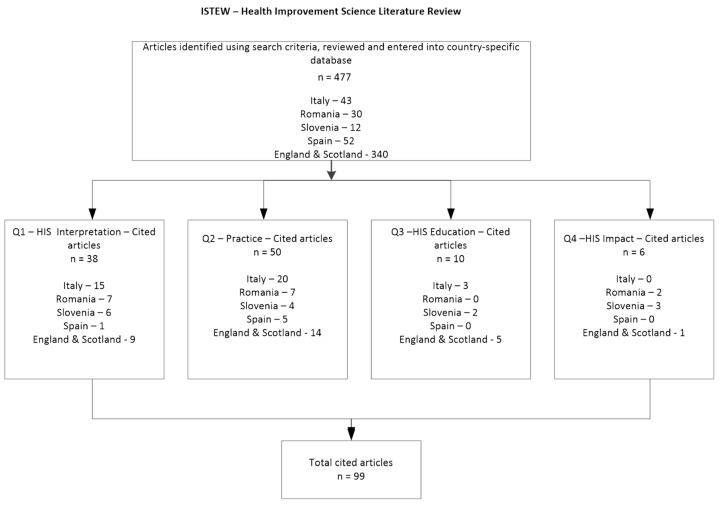
Criteria for inclusion.

Articles chosen for further review were selected by ISTEW researchers (except Poland, not participating in this work package), all of them experienced in HIS and quality improvement, based on their relevance to the field and research quality, as can be seen in Figure 1. However, because of the different healthcare systems of each country, views on relevance may to some extent be context specific. 

A further consideration was that of variation in the range of references across the different countries and research questions. In England and Scotland, an initial search yielded over 4000 references, whereas, in Slovenia, the first search yielded only thirty-four articles, of which only twelve were selected for further review. In England and Scotland, work was carried out jointly, which enabled the teams to have access to two sets of library databases, increasing the scope and enabling each team to look in greater depth at two of the four research questions. In Romania and Spain, the search strategy was adjusted to take account of the fact that more country-specific articles could be found in local databases (such as the bibliographic repertory of the national library). In line with this, the search terms used in the literature review were selected throughout the partners’ consensuses, according to their own experience, using the keywords that currently were referred to HIS and were utilized as synonyms in their own countries and in their mother languages (Table 1).

## 3. Results 

The consensus responses to each of our four key research questions are set out below.

### 3.1. How Is Improvement Science Interpreted?

An over-riding theme was the fragmentation, diversity, and rapidly developing view of improvement science in the field of healthcare and service providers.

In England and Scotland, commentators noted that it was difficult to identify a single, agreed upon definition of improvement science, as the field was “complex and continually evolving” [15]. A number of terms have been used to describe HIS, including “implementation science, the science of improvement, improvement science, translational research, translational science, measurement for improvement, quality improvement methods, quality improvement science, the science of quality improvement, evidence-based practice, knowledge translation and research utilisation” [16].

All partners reported the lack of a standard definition for HIS in their literature, with, for example, one UK article reporting on 47 different models for knowledge translation in a healthcare environment [17].

Using agreed search terms (Appendix A), each partner team reached consensus from the themes that came up from their individual searches in their own nation and specific contexts. For example, in Romania, the key theme was the transition at the national level from a communist (i.e., centralised, with little individual responsibility) to a post-communist society, with its focus on personal accountability and the importance of teamwork [18,19]. Italian literature tended to portray HIS as a set of tools and techniques to solve particular problems, rather than as a generalizable theory, discipline, or science. Examples included strategies to improve organization performance and the overall quality of care, in terms of medication or communication with clients/patients [20,21,22,23,24,25,26,27,28,29,30,31,32,33,34].

In Slovenia, the need for more HIS research, particularly in the field of nursing, was highlighted [35]. Quality assurance was not yet viewed as a major concern in the Slovenian healthcare system and managers were said to lack information and guidelines on the subject [36]. In Spain, articles tended to focus on specific healthcare issues regarding quality of care and quality improvement, such as clinical hand-washing, disease prevention, and patient safety checklists. 

In a separate ISTEW work package (Table 1), colleagues working on a review of available education found that keywords appearing in the titles of courses varied from country to country, perhaps reflecting current ‘priorities’ for improvement inside the education field [8]. Table 1 shows the frequency of keywords per country found in relation with HIS education. This variety of terms suggests the evident lack of consensus and standardization of the concept of HIS in the participating countries. 

HIS is interpreted differently in each European educational system, as shown, however, one thing in common in all searches is the lack of professional values linked to HIS understanding and interpretation. Values, such as person-centred care or compassionate care should be also associated HIS as a core component of it instead of just the traditional quality management perspectives [37].

### 3.2. How Is Improvement Science Practised?

Partners’ narratives revealed a wide range of settings in which HIS tools and techniques were practised, but, as with the definition, these did not link back to a common interpretation or agreed way of working. There were, therefore, many examples of specific improvement projects and their evaluations, but fewer narratives about the wider-scale adoption of improvement tools and techniques. This perhaps reflects Perla’s view [15], that the healthcare system cannot be seen as a machine, but as embodying, emerging, and recursive processes that are only now beginning to be understood. 

Storey [38] describes how public concern and the increasingly strong policy emphasis on quality and the safety of patients in acute settings has led to a proliferation of healthcare quality initiatives in the UK context. Alexander et al. [39] reviewed 107 studies, examining the implementation of quality improvement interventions, identifying that organizations often struggle with implementation. They concluded that most studies focused on the internal context and organizational processes, whereas the external context, as well as organizational processes, most strongly affected implementation.

There have been calls for the broad implementation of translational research in public health [40], especially for the care of older people and for evidence-based practice in the applied social sciences [41,42]. However, across all disciplines, there is limited scientific evidence of successful implementation available in the literature, and there exists considerable discussion of barriers. As O’Donnell stated in her PhD research, “the natural intensity and busyness of the healthcare system often acted as natural barrier to staff engagement with quality care” [43].

In Italy, patient safety was highlighted as a major concern, particularly in relation to surgical infections and the administration of medication [29,30,31,32]. Benucci et al. [44] evaluated medical error in terms of claims for damages, and Salmoiraghi et al. [45] focused on surgical infections. Other studies on client/patient satisfaction found that a more person-centred attitude on the part of healthcare professionals can have a positive effect on treatment compliance [46,47,48,49].

In Romania, organisational and national policies in relation to healthcare improvement were paramount. Overall, HIS was said to be limited by lack of resources, with only 5.9% of GDP spent on healthcare compared to an average in western Europe of 9–12% [50]. The small number of practising physicians has also led to lower health outcomes compared to western European countries [51].

In Slovenia, Robida [52] described work on the management of health institutions and health professionals to improve knowledge and the implementation of medical treatment and patient care, aiming to include both healthcare professionals and patients. The importance of nurses being aware of processes and working on continuous improvement is also stressed by Donik [53].

Spanish literature focused on advancements in healthcare by improved procedures and processes (reducing mistakes in clinical settings and through e-health technologies) and strengthened healthcare policies, clinical practice guidelines, and clinical protocols [54]. The patient safety approach is mainly how HIS is understood and practiced within the Spanish context, though some references were found regarding the practice of healthcare improvement in cooperation projects developed in low-income countries by Spanish authors [55,56]. Furthermore, efforts have been made in the Spanish context to create an evaluation framework for HIS training in practice [5].

The variation in the way that HIS is approached in the partner countries’ literature reflects discussions within the project group when trying to agree a consensus definition of HIS. Partners in Slovenia and Spain for example tended to stress the ‘science’ aspect of the subject, with its corresponding need for clarity and evidence-based practice. In Italy, the main focus was on efficiency and quality assurance, whereas in Scotland and England the impact on the patient and the importance of careful implementation were key themes [11].

In the wider European community, the contribution of HIS to practice has been developed in recent years. For example, in Sweden, we found several recommendations for practice regarding scale-up improvement and improvement leadership [57,58]. Also, annually, the Quality Improvement Research Network (QIRN) organises scientific meetings to discuss recent advancements in quality improvement within Europe. 

### 3.3. How Are Multidisciplinary Health Students Educated in (the Various Components of) Improvement Science?

Compared to results from the previous two questions, partners reported a paucity of literature directly concerned with this topic regarding teaching methods, the educational setting, the content of curriculum, etc., linking to HIS. In the UK, only five texts were identified, four of which related to the way in which multidisciplinary students from different health fields were currently educated. Four references were found in Slovenia and Romania, and three in Italy. Most information in this part of the article is therefore drawn from a further two ISTEW work packages. One of these was led by partners in Romania, who led a more in-depth analysis of existing training and education for HIS involving web and database searches and direct contact with educational institutions [8]. The second work package carried out a gap analysis between the need for and provision of HIS education in partner countries [9].

Common themes included empowerment—the need to follow through on the interest of healthcare professionals through formal and on-the-job training, and the need for further research to demonstrate the link between effective training and improved healthcare outcomes that affect the quality of patient care.

**Figure 2:**
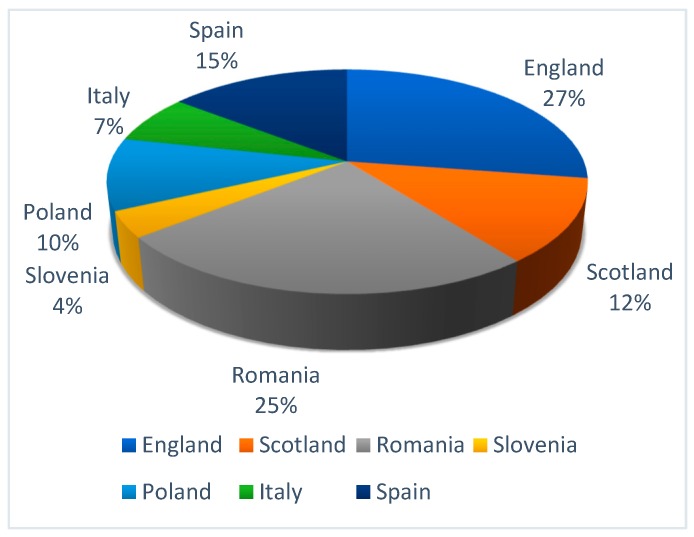
Relative distribution of courses with an element of healthcare improvement science (HIS) across the partner countries.

The number of courses with an HIS component varied from 66 (England) to 7 (Poland). In England, Scotland, Romania, Poland, and Italy, these courses were open to non-medical students, whereas in Slovenia and Spain they were not. All of the courses were EQUAR (European Quality Assurance Register for Higher Education) quality assured, except those in Slovenia. Figure 2 shows the relative distribution of courses with an element of HIS across partner countries [8].

Colleagues from Poland working on a gap analysis work package concluded that although there was a broad range of courses with a HIS element, HIS training, as a whole, was in its infancy across most of Europe. Most of the courses were at a postgraduate level, and few had been systematically evaluated [9].

This is also reflected in our literature review findings. In Italy, Napoli et al. found that whilst nursing students were interested in HIS, their knowledge of the subject was weak. In Romania, all medical schools have modules, and, in some cases, Masters programmes that involve contents indirectly related to improvement science, including health management, bioethics, and health policy [48]. In Spain, the most common HIS keywords were “evidence-based practice”, “translational research”, “quality improvement” and “patient safety”. In Slovenia, a postgraduate multiprofessional curriculum on patient safety was introduced for the first time at the College of Nursing in Jesenice, Slovenia. A knowledge evaluation conducted in students after attending 11 modules on patient safety showed that there were no statistically significant differences between their knowledge on 10 topics of patient safety before and after teaching [52].

In the UK, McGaghie et al. found that simulation-based mastery learning in medical education can produce downstream results, demonstrating that the new discipline of implementation science holds promise to explain why medical education innovations are adopted slowly [59]. Jones et al., described how two universities in Wales are working in partnership with NHS organisations and the Healthcare Improvement Open School to integrate quality improvement into undergraduate nursing programmes [60]. Brody et al. suggested that greater emphasis needs to be placed on disseminating existing evidence-based care and ensuring that programmes are interprofessional in nature [61]. However, Robinson et al. concluded that courses to build interprofessional skills, whilst valuable, pose administrative and educational challenges to development [62]. 

Since 2016, nursing students from different European countries have been educated in specific HIS education, through the International Summer Programme on HIS Introduction and Immersion, conducted by the University of Alicante, Spain: https://web.ua.es/es/verano/documentos/2019/dipticos/immersion.pdf [7]. In addition, one PhD student, from the Faculty of Health Sciences at the University of Alicante, working specifically on HIS, has been identified with a proposal entitled: “From theory to practice, developing a global healthcare improvement science culture”, and a PhD dissertation on HIS has been also produced at the University of the West of Scotland by one the ISTEW researchers [43].

### 3.4. What Is the Impact of Education in Improvement Science on Service Provision?

A key theme was the recognition that Healthcare Improvement Science education was still in its infancy, with no consensus or clear guidelines, therefore, it is difficult to describe the impact it has on the healthcare system as a whole. Italy, Romania, and Spain could not find any articles directly relating to the impact of HIS education on service provision. In Slovenia, only two references by Slovene authors were found, both emphasizing the importance of linking evidence-based knowledge to healthcare policy.

However, tangible examples were found in the UK. Such was the case of a Scottish patient safety programme, where training and project work were able to deliver practical benefits. For example, a project to reduce hospital associated infections in intensive care was able to demonstrate a reduction of two days in the average length of stay [63]. A similar initiative, the Health Foundation’s Safer Patients programme helped 24 organisations to build a safer NHS [64]. In other countries, such as Ireland, the impact of HIS has been linked with values such as compassion in recent years. Such was the case of the “Productive Ward: Releasing Time to Care” (PW) project, which was a quality improvement programme designed to meet the value and mission intentions around quality and compassion. They measured the impact that the PW had on direct patient care times and the capacity of ward-based teams to provide compassionate care [65]. In Belgium, such impact has been recognized recently, through the first European Magnet-Hospital Accreditation for UZA Hospital in Antwerp [66].

## 4. Discussion

In this article, we described the methodology carried out to identify how the status of healthcare improvement science culture is understood in Europe, how it is practised and taught across European healthcare and higher education institutions, and the outcomes of applying such a methodology in practice. In fact, the findings of this literature review contributed to the development of a HIS consensus definition, 4 HIS education modules, and a HIS evaluation framework [3,5,6].

The project found clear differences in healthcare contexts and understandings of HIS, but it also uncovered its potential for increasing patient safety and satisfaction. Whilst languages may differ, the healthcare improvement challenge remains the same, namely, elucidating how to improve the quality and reliability of patient care, reduce avoidable mistakes, and improve the effectiveness of the healthcare system. 

This literature review has indicated that European education and implementation of HIS is still in its infancy in most countries. If we compare the status of healthcare improvement science education in the European continent with that of the United States of America, the gap is more evident. In the USA, HIS has been gaining momentum since the 1980′s, though focusing on management primarily. The Institute of Health Improvement (IHI) was officially founded in 1991, although its work began in the late 1980s as part of the US National Demonstration Project on Quality Improvement in Health Care, led by Dr. Don Berwick and a group of visionary individuals committed to redesigning healthcare into a system without errors, waste, delay, and unsustainable costs. Since then, they have grown from an initial collection of grant-supported programs to a self-sustaining organization with worldwide influence. Today, the IHI is an influential force in health and healthcare improvement in the US, and has a rapidly growing footprint in dozens of other nations, especially English-speaking ones and others which mainly follow US publications, including Canada, England, Scotland, Denmark, Sweden, Singapore, Latin America, New Zealand, Ghana, Malawi, South Africa, the Middle East, and elsewhere [67]. In the UK nowadays, organizations such as “The Health Foundation” are working to improve healthcare outcomes in the country, developing many specific HIS initiatives, education, and research, as shown in the literature review conducted, especially in Scotland [68]. 

## 5. Discussion of Limitations

Although a structured methodology was adopted, it became increasingly obvious that subtleties and nuances in translation revealed problems in the transferability across the five languages of the study. The result of this is that our findings are more interpretive and narrative than was anticipated, as in many countries the main results were related with synonyms, not directly with the healthcare improvement science term. However, the review was then supplemented by an interactive consensus session at our conference in Bled, Slovenia, which enabled us to recognise tensions between our various contexts and to increase shared understanding. 

It should also be noted that the review was only related to those countries taking part, thereby missing out on major healthcare systems from other European nations, although we have outlined published initiatives by authors from Sweden [57], Ireland [65] and Belgium [66], 

What has emerged from this status evaluation through the literature review is that it is complex to define healthcare improvement science or describe its implementation in our partner countries in a standardised manner [3]. Also, because of the relative difference in the development of thinking across partner countries, not all partners were able to find literature to support all of the four research questions, particularly those relating to educating for and the impact of HIS. For the purposes of this article, we have therefore included work from other ISTEW work packages that have enhanced our consensus statement on its status in Europe, giving us more detail than we were able to access through literature review searches alone, particularly in relation to education. The unpublished reports we draw on here are: The definition of HIS [11]; Clarification of Competencies and Capabilities [10], and Review [8] and Gap Analysis [9] of Available Education/Training. The work packages on which these reports are based used a variety of methods, including questionnaires, web-based research, interviews, and a modified Delphi approach.

## 6. Conclusions

The partners in this project acknowledge the benefits of healthcare improvement science as a core competency for healthcare professionals’ education in Europe. From the authors’ experience, European projects such as ISTEW are needed, where they help to develop a better understanding between educational and healthcare systems in different countries, and additionally to reach agreements on how new educational perspectives should be implemented into healthcare practice, research, and policy.

Building on the reports referred to in this article, the ISTEW project has produced the following outcomes: A healthcare improvement science definition, teaching modules regarding HIS, and a HIS evaluation framework [3,5,6]. In addition, further efforts have been made to disseminate and put the first step to developing a culture of healthcare improvement science forward, through the ISTEW project outcomes produced until now, as for example with an International Summer School named as “Immersion to Healthcare Improvement Science” that takes place in the Faculty of Health Sciences at the University of Alicante, one of the ISTEW partner teams, every year since 2016 [7]. Across these outcomes, healthcare improvement science education and its implementation into practice could be increased, building towards a substantial increase in HIS awareness across Europe, promoting new initiatives.

The literature review presented highlights the dispersed understanding and practice of healthcare improvement science across Europe in the academic and clinical fields nowadays, which justifies the need of more specific efforts. Our findings have brought to light the gap existing in HIS-related theories and sources that are applied into real contexts within European countries where healthcare systems and educational institutions are, far from other similar initiatives developed in other parts of the world, such as the USA, where health systems’ principles determine a different HIS understanding. Future approaches in Europe should concentrate on exploring how the context and culture affect the success of implementing HIS initiatives, as it has been demonstrated through the review that HIS is not understood and practiced in the same way in all countries. Moreover, the return on investment (ROI) of such educational interventions into the health systems should be considered too. ROI is not only about return in terms of economic aspects, but also about evaluating to which extent HIS is understood and implemented by healthcare workers, including concepts such as the caring values and social contribution linked to those activities. Focusing on the previous step of healthcare professionals, undergraduate healthcare studies should also be included in this evaluation. Those students, as future professionals, are very well-positioned to play pivotal roles in the conversion of our healthcare systems to ones that incentivize high value, patient-centred care, improvement, and compassion. 

Investing in a better-educated professional staff regarding the scope of healthcare improvement science could improve the quality of patient care and the development of healthcare systems.

## Figures and Tables

**Table 1:** Keywords frequency per country.

Country	Frequency				
	Leadership	Improvement	Management	Healthcare Studies	Evidence Based Practice
England	18/66				
Scotland		2/8			
Romania			9/60		
Italy			7/16		
Poland			10/27		
Slovenia				4/9	
Spain					20/35

## Appendix A. Search Terms and Research Protocol

Following some experimentation and discussion between library and project staff at both sites, the English team agreed on the following keywords and search terms:

**Question 1—Interpretation**

Improvement N2 science OR “translation * research” OR “translation * science” OR “translation * knowledge”
OR Implement N2 science (in abstracts and titles)
AND
Interpret * OR defin * OR framework * OR theor * OR technique* OR tool * OR method *

**Question 2—Practice**

Improvement N2 science OR “translation * research” OR “translation * science” OR “translation * knowledge”
OR Implement N2 science (in abstracts and titles)
AND
Practic * OR impact * OR implement * OR deliver * OR integrat * OR embed * OR “service provision” OR application *

**Question 3—Education of students**

Improvement N2 science OR “translation * research” OR “translation * science” OR “translation * knowledge”
OR Implement N2 science (in abstracts and titles)
AND
Education * OR curriculum OR training OR course * OR facult *
AND
Student * OR cohort *

**Question 4—Impact of Education on Service Provision**

Improvement N2 science OR “translation * research” OR “translation * science” OR “translation * knowledge”
OR Implement N2 science (in abstracts and titles)
AND
Education * OR curriculum OR training OR course * OR facult *
AND

**“Service provision” OR service N2 improvement OR outcome***
